# The Effectiveness of Zero-Day Attacks Data Samples Generated via GANs on Deep Learning Classifiers

**DOI:** 10.3390/s23020900

**Published:** 2023-01-12

**Authors:** Nikolaos Peppes, Theodoros Alexakis, Evgenia Adamopoulou, Konstantinos Demestichas

**Affiliations:** 1 School of Electrical and Computer Engineering, National Technical University of Athens, 15773 Athens, Greece; 2 Department of Agricultural Economy and Development, Agricultural University of Athens, 15855 Athens, Greece

**Keywords:** zero-day attacks, malware detection, Generative Adversarial Networks (GANs), cybersecurity, deep learning, information security

## Abstract

Digitization of most of the services that people use in their everyday life has, among others, led to increased needs for cybersecurity. As digital tools increase day by day and new software and hardware launch out-of-the box, detection of known existing vulnerabilities, or zero-day as they are commonly known, becomes one of the most challenging situations for cybersecurity experts. Zero-day vulnerabilities, which can be found in almost every new launched software and/or hardware, can be exploited instantly by malicious actors with different motives, posing threats for end-users. In this context, this study proposes and describes a holistic methodology starting from the generation of zero-day-type, yet realistic, data in tabular format and concluding to the evaluation of a Neural Network zero-day attacks’ detector which is trained with and without synthetic data. This methodology involves the design and employment of Generative Adversarial Networks (GANs) for synthetically generating a new and larger dataset of zero-day attacks data. The newly generated, by the Zero-Day GAN (ZDGAN), dataset is then used to train and evaluate a Neural Network classifier for zero-day attacks. The results show that the generation of zero-day attacks data in tabular format reaches an equilibrium after about 5000 iterations and produces data that are almost identical to the original data samples. Last but not least, it should be mentioned that the Neural Network model that was trained with the dataset containing the ZDGAN generated samples outperformed the same model when the later was trained with only the original dataset and achieved results of high validation accuracy and minimal validation loss.

## 1. Introduction

The digitization of everyday activities has led to a wide variety of computer and smartphone applications and systems that rely on Internet connectivity. Moreover, the rapid evolution of the Internet of Things (IoT) paradigm in almost every domain, ranging from agriculture to industry and health services alongside the advantages it offers, also leads to numerous vulnerabilities. In this light, a major concern of cybersecurity experts are the zero-day (0-day) vulnerabilities, attacks, and exploits. As defined by Kaspersky [1], zero-day vulnerabilities are software weak spots that are detected by attackers before the vendor of the software detects and patches them. An exploit is the method an attacker follows to take advantage of the unpatched vulnerability, and lastly, an attack is defined as the utilization of the exploit by a malevolent party with the purpose of causing damage to the software vendor or the software users [1].

Zero-days vulnerabilities reside in almost every software and hardware and in most cases are patched after their detection. For example, zero-day hacks can take place in operating systems, open-source libraries and tools, hardware and firmware of certain devices, web browsers and plugins as well as widespread applications such as office and photo/video editing applications. The attackers exploiting zero-day vulnerabilities can be cybercriminals who want to damage an organization or a country, individuals aiming for personal gain, or hacktivists mainly acting on social and political motives or even employees involved in corporate antagonism. The motives of the attackers as well as the nature of the attacked infrastructure define the victims of each of these attacks. Thus, there is a wide range of potential affected users. More specifically, when a zero-day attack is performed to a common piece of software, such as browsers, operating systems, or office applications, then the affected users are individuals who use the software. However, when there are political or social motives as well as corporate espionage reasons, the victims may include entire countries, social networks, government agencies and large organizations. Based on the nature and the motives of an attack, zero-day attacks can be characterized as targeted and non-targeted ones. Targeted attacks focus on specific users of organizations whilst non-targeted ones aim to attack whoever uses a compromised component, thus affecting as many users as possible. Either way, exploited zero-day vulnerabilities can lead to financial damage of millions of dollars or euros.

Google’s Project Zero team reported, in the first half of 2022, that 18 different zero-day attacks were detected, nine of which were variants of previously patched vulnerabilities [2]. Additionally, according to the Mandiant report of 2021 [3], zero-day exploits over-doubled compared to previous years. One can assume that zero-day vulnerabilities increased as there are more and more tools and applications that are exposed to malicious enablers. A well-known indicative example of zero-day attack is the Microsoft CVE-2016-0167 vulnerability [4], which enabled threat actors to gain SYSTEM privileges on Microsoft Windows machines causing significant problems to users. Another infamous zero-day attack is the Aurora operation, which targeted several different organizations such as Yahoo, Symantec, Google, Microsoft, Morgan Stanley and more and affected millions of users [5]. Other recent known zero-day attacks include the Zoom vulnerability [6], which enabled malicious actors to execute code remotely via the Zoom application, the iOS zero-day vulnerability discovered in the roll-out of iOS version 16.0 [7] as well as the Chrome bug in 2021, which forced Google to immediately roll-out a security patch [8].

The aim of this study is to examine software zero-day exploits data in order to generate new synthetic instances of such data using a Generative Adversarial Network (GAN) architecture. These new data will be used as input to a Deep Learning (DL) based detector of zero-day exploits, the performance of which will be evaluated both with and without transfer learning features. In this way, the added value of this study lies on two main aspects: Firstly, to create and enhance data generation capabilities concerning software zero-day exploits and secondly to assess the capabilities of DL systems to detect and prevent zero-day attacks. Thus, in the context of this study, a new approach for tackling zero-day vulnerabilities is proposed both in terms of data generation as well as detection by means of deep learning. The purpose of the study is to lift the limitations caused by the lack of data in the field of cybersecurity, especially regarding zero-day attacks. Thus, by studying and confirming the efficiency of GANs for data generation, ML and DL algorithms and tools can be trained more efficiently since the synthetic data generated by GANs can improve the performance and credibility of such systems. This will lead to better cybersecurity solutions which will ensure the safety of interconnected infrastructure and minimize damage costs.

The remainder of the paper is organized as follows: Section 2 presents related works, focusing on the domain of zero-day vulnerabilities, exploits and attacks. Section 3 describes the methodology designed and developed, whilst Section 4 elaborates on the evaluation of the usefulness of the generated dataset in the relevant Deep Learning (DL) classifier. Finally, Section 5 draws interesting conclusions.d

## 2. Related Works

The continuous increase of system damages due to malware and zero-day attacks led to the need for studying, in greater depth, zero-day detection methods. Thus, several such studies have been conducted and can be found in the relevant literature. Considering the two main pillars of the present study, as described above, related works presented in this section are mainly focused on zero-day attacks’ data generation and classification.

Jin-Young Kim et al. [9] propose a malware detection system architecture that uses input real and fake malware data with modified features (compared to the real ones), generated from a deep-convolutional generative adversarial network (tDCGAN) based on a deep autoencoder (DAE) that achieves higher accuracy and learning stability in comparison to similar models. In their study, the authors evaluate the performance of the tDCGAN for generating zero-day malware attacks data by adding noise using GANs [9]. The performance evaluation on zero-day attacks only focuses on the initial training of each model and the models are not retrained with newly generated samples in order to compare the training process as described later in the present paper. In the scope of zero-day attacks data generation, Dong-Ok Won et al. [10] suggest a framework (PlausMal-GAN with representative GAN models) developed and trained in order to produce analogous zero-day malware images with high quality and diversity. These generated images are used as training input to the discriminator of the GAN model in order to increase the detection ability of several types of analogous zero-day malware attacks. The main difference of this approach compared to the present study is that the effect of the augmented dataset compared to the original one was not evaluated. Furthermore, Tram Truong-Huu et al. [11] studied the application of GANs in the domain of network anomaly detection. Multiple datasets are adopted during the experimental stage in order to evaluate the performance of GANs compared to other methods used for network anomaly detection. The set of experiments performed during this study demonstrates important improvements compared to existing DL techniques for network anomaly detection, in terms of various metrics of accuracy and performance. This approach could be considered an effective step in detecting unknown anomalous behaviors or zero-day attacks. Rodolfo Valentim et al. [12] propose a GAN architecture in order to study and tackle the problem of phishing squatting detection, which, in the future, could also serve as the starting point for solving the issue of zero-day phishing attacks. However, despite the very interesting concept, their approach was not adequately explored so as to reason on the use of GANs for the intended purpose. Moreover, a multistage multi-class classifier based on semi-supervised GANs is developed by Santosh Kumar Nukavarapu and Tamer Nadeem [13,14]. Automatic feature extraction is performed using minimal labeled data while achieving high predictions results in terms of accuracy. For example, the classifier developed in that study can infer the device type (IoT, Non-IoT, and anomaly) of any new device with 90% accuracy. In the same study, the ability of the trained model to support novelty feature detection, such as zero-day malware attacks, is also demonstrated. The main difference of this approach compared to the current study is that focus is given to the classification of hardware anomalies that may occur in an IoT network and not software vulnerabilities. Santos et al. [15] study and propose a sequence of opcodes in order to add flexibility that enables capturing zero-day attacks. Additionally, Huda et al. [16] propose a semi-supervised learning model that receives unlabeled data as input and results in the integration of knowledge about unknown malware data in an automatic way. This is accomplished through the use of the unlabeled data in combination with the integration of the k-means clustering algorithm and the Support Vector Machine (SVM) classifier based on Term Frequency–Inverse Document Frequency (TF-IDF) features. Their solution, a predecessor to other approaches presented above, did not, however, engage GANs. Pandey et al. [17] present a similar approach to the present study by engaging a GAN to generate attack data in order to evaluate the performance of a Decision Tree (DT) classifier. Through their study, Pandey et al. highlight the effectiveness of the GAN to generate attack samples that are very similar to real ones and then examine the way the performance of the DT classifier is affected when those samples are ingested into the testing dataset. Comparing the study by Pandey et al. with the methodology presented in this study, the main differences lies on the type of classifiers used in each study (DT in case of [17] and NN in our study) as well as on the different use and evaluation of the synthetically generated data by GANs. In [17] the authors claim that the already trained DT classifier performs worse when the augmented data are ingested in the training whilst in the present paper, as shown in Section 4, the behaviour of the NN classifier is evaluated when the augmented data are ingested both in the training and the testing dataset. Additionally, Shu et al. [18] designed and developed a GAN model using a Variational Autoencoder (VAE) that proved very efficient for bypassing an Intrusion Detection System (IDS). Further, in [18], the authors focus on the efficiency of a properly trained GAN to bypass IDS.

Moving further from zero-day data generation and exploration using GANs as presented in the previous paragraphs, other studies can be found that place their main effort on the detection and classification of zero-day attacks. In this light, Zhou and Pezaros [19] examine and evaluate six different machine learning models for zero-day intrusion detection. Their study indicates that the Decision Tree (DT) algorithm achieves the best result in terms of accuracy (96%) and false positive rate (5%). The six different classifiers were evaluated using a modified version of the CIC-AWS-2018 intrusion dataset that consists of eight different types of zero-day attacks. Their approach focuses solely on the evaluation of different ML classifiers by training them with the CIC-AWS-2018 dataset and not on exploring the role of GANs for data augmentation. Moreover, the models were not evaluated in completely unknown threats such as those produced by a GAN, mimicking the authorship of zero-day threat. Bilge and Dumitras [20] propose addressing zero-day attacks by examining and identifying their characteristics before and after an attack takes place. Their study indicates that, once a vulnerability is revealed, the chance of its exploitation to occur increases five times. Additionally, the authors report that a zero-day attack can last approximately for 312 days and at most 30 months. Last but not least, the team suggests a heuristic approach for zero-day attacks identification [20]. For detection and classification purposes of zero-day attacks, Alazab et al. [21] propose and develop a machine learning framework in order to detect a zero-day vulnerability. In their study, eight different ML classifiers are trained and evaluated based on API call sequences and frequencies. More specifically, the proposed framework first retrieves the API call sequences from the executable files, then updates the signature database based on these API calls and finally reports the similarity value. The classification task is performed using a supervised learning algorithm with eight classifiers, namely: Naïve Bayes (NB) Algorithm, k—Nearest Neighbor (kNN) Algorithm, Sequential Minimal Optimization (SMO) Algorithm with 4 different kernels (SMO—Normalized PolyKernel, SMO—PolyKernel, SMO—Puk, and SMO—Radial Basis Function (RBF)), Backpropagation Neural Networks Algorithm, and J48 decision tree. The SMO—Normalized PolyKernel algorithm achieves the best results in terms of true positive and false positive rates, 98.6% and 2.5% respectively using Support Vector Machine (SVM) for the detection of unknown malware samples with obfuscated code [21]. Comar et al. [22] present a framework for zero-day malware detection that leverages the accuracy of supervised classification methods in detecting known classes, combined with the adaptability of unsupervised learning for the same purpose. Their framework consists of six different modules, namely: (1) A data capture module, (2) an intrusion detection/prevention system (IDS/IPS), (3) an information storage module, (4) a feature extraction and transformation module, (5) a supervised classifier, and (6) a user interface. This framework engages a class-based profiling technique which enables distinction of unknown and known malware and is then able, using network traffic features for the SVM classifier, of detecting zero-day malware [22]. Sharma et al. [23] also propose a zero-day attack prevention strategy, called Distributed Diagnosis System (DDS), based on context graph strategy. Their solution consists of three parts, namely: (1) The Central Diagnosis System (CDS), (2) the Local Diagnosis System (LDS), and (3) the Semi Diagnosis System (SDS). The purpose of the DDS system is to guarantee the safety of IoT devices against possible zero-day vulnerabilities. Sharma et al., based on their findings, suggest that it is important to immediately remove a compromised IoT device in order to mitigate the cascading effects and avoid infection of the other IoT devices on the network [23]. An interesting PhD thesis with a different aim was performed by Michael Glen Miller [24]. Miller forms and examines research questions concerning the adoption of efficient machine learning solutions for zero-day attacks detection. Additionally, Miller explores the application of feature selection techniques for zero-day malware data and suggests that the combination of a reduced number of features can lead to better results based on the classifier. Moreover, the ensemble of different classifiers both for classification and clustering applications can lead to improved results regarding of zero-day malware attacks. Studies [19,20,21,22,23,24] mainly focus on ways to detect different forms of zero-day vulnerabilities and attacks such as malware, API calls sequence, IoT compromised devices, etc. The results of these studies are encouraging and promising and pave the way for further exploration in this domain. Building on these approaches, this study aims to expand current solutions in a way that adds value both in the direction of data generation of zero-day attacks as well as in the detection of them. The first part of the present study i.e., data generation is of utmost importance as most solutions and approaches underline the lack of data availability. Additionally, data availability is directly affecting the performance of detection methods that are based on ML and DL techniques.

## 3. Data Feature Selection and Generation Using GANs

### 3.1. Initial Dataset and Features

**Figure 1:**
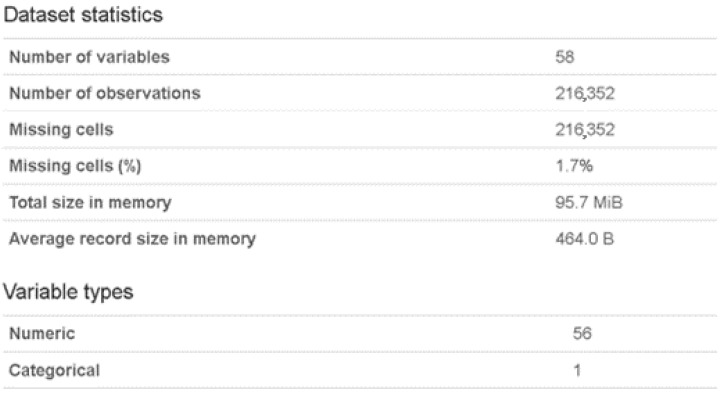
Initial dataset statistics.

The original dataset that was used for the purposes of this study was obtained from the Kaggle Website [25], a well-known repository of open datasets. Based on Kaggle’s information on this dataset, the raw data have been obtained from the malware security partner of Meraz’18, the annual techno-cultural festival of IIT Bhiali. The dataset contains two parts: namely the training data and the evaluation data. More specifically, the training data include 216,352 records from which 140,849 records are labelled with “0”—malware-whilst the remaining 75,503 are labelled with “1”—legitimate. The evaluation data consists of 88,258 records without label. The training data contain 57 (including the ID of each sample) features which are, as described in paragraph 3.2, reduced for the purposes of this study. Some statistic details of the dataset can be found in Figure 1.

The considered dataset is a dataset widely-used for evaluation purposes, thus covering the needs of this very study. The preprocessing and feature selection procedures are presented in detail in the next paragraphs of the current section. Additionally, Table A1 in Appendix A presents the data type of each of the 58 features of the initial dataset.

### 3.2. Feature Selection

One of the most important stages, before the training and evaluation procedures of a deep neural network take place, is the feature engineering (pre)processing (or feature selection). Feature engineering is the task of selecting the required independent features of the (initial) dataset in order to improve the overall performance of the predictive model [26]. This kind of processes require high computational costs in terms of hardware resources. The different approaches used to automate this process rely on feature selection based on the explicit expansion of the provided datasets following the feature transformation as well as on their characteristics’ exploration through their evaluation-guided search [27]. Selecting the most relevant features is an important step during the preprocessing stage of a large dataset that will be later used for training pursposes. It assists in eliminating less important parts of the data, thus reducing the cost of resources and training time. Some of the most popular techniques for feature selection are presented below [27]:

- Univariate Feature Selection. This method applies statistical tests to the provided dataset in order to extract the strongest correlation between the independent features and the selected output feature. One of the most commonly used methods, which involves a variety of statistical methods, is the SelectKBest method [28].
- Extra Trees classifier Methods. In the context of this method, an importance score of each independent feature to the one defined as dependent is extracted. The higher the score, the more relevant the feature towards the target/depedent variable.
- Correlation Matrix with heatmap, where a 2D (two dimensional) data representation is acquired. This kind of diagram depicts the relationship between the dependent and the independent features of the-under study-dataset, where a correlation scale is also provided (and visualized) through the integrated heatmap.

During the current study, the first steps of the feature engineering process included the identification and the removal of the n/a (non-available) values in the provided dataset as well as the recognition and the transformation of the categorical features after the application of the label encoder [29] to the corresponding values of the initial dataset. The next steps included the application of the three previously mentioned (feature selection) methods separately, in order to identify the most relevant features to the independent one (‘legitimate’ feature column).

Subsequently, the first feature selection method that was applied on the dataset was the Univariate Feature Selection, and specifically, the SelectKBest method. Τhe feature selection model was then defined after using the SelectKBest class. For classification purposes, the ‘f_classif’ method [30] was selected as the scoring function and the target number of the top extracted features were defined after setting the k parameter equal to thirty (30). Table 1 depicts the top 30 features of the initial dataset based on their importance score following the analysis of the UVA feature selection (SelectKBest). It is clear that the most relevant feature to the independent one seems—for the specific method—to be the so-called ‘SectionsMaxEntropy’.

**Figure 2:**
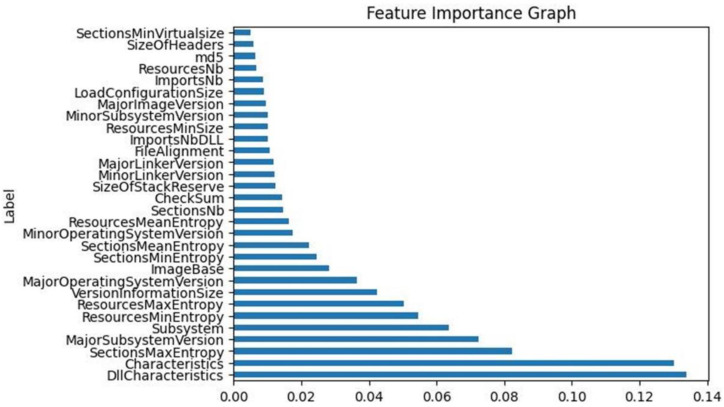
Top 30 features of the initial dataset based on the feature importance.

Then, and in order to select a set of the most relevant features to maintain in the final dataset, the Extra Tree Classifier method was applied [30] so as to compare the results to the ones produced by the SelectKBest method. Figure 2 visualizes the extracted results of the feature importance for the top thirty features of the dataset in correlation with the output target of the ‘legitimate’ feature. It becomes clear that the most important data feature—using the Extra Tree Classifier method—is the ‘DllCharacteristics’, whereas the least important one is the ‘SectionsMinVirtualsize’. Additionally, it was observed that the top eight features depicted in Figure 2 were also the top eight most relevant features in Table 1.

Consequently, following and combining the results of each method presented and applied separately on the initial dataset, the eight features that were selected for the creation of the smaller dataset, aimed to be provided as input to the GAN network, so as to generate similar-looking tabular data, are namely:

1. DllCharacteristics
2. Characteristics
3. SectionsMaxEntropy
4. MajorSubsystemVersion
5. Subsystem
6. ResourcesMinEntropy
7. ResourcesMaxEntropy
8. VersioninformationSize

The aforementioned eight features that were selected are those with the higher importance based on both feature selection methods applied independently.

**Figure 3:**
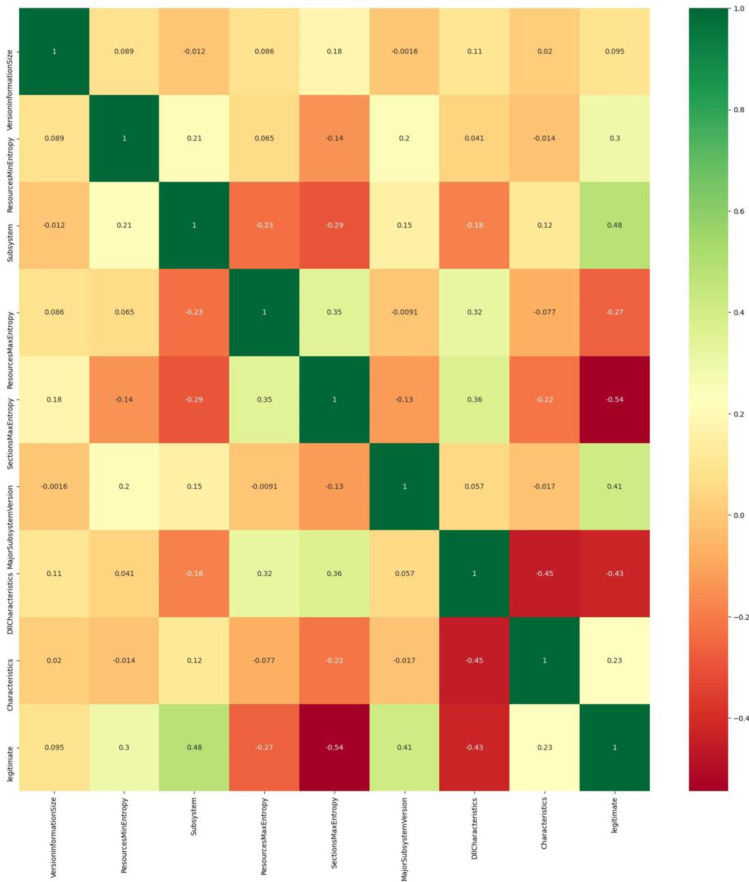
Correlation matrix of the final selection of features from the initial dataset.

Figure 3 depicts the correlation matrix with the integrated heatmap, a table that displays the correlation coefficients for the selected eight variables of the final dataset. As discussed, the correlation matrix depicts the correlation between all possible pairs of values. By examining the extracted results, it is clear that the most relevant feature (between the selected ones) is the ‘Subsystem’, while the ‘SectionMaxEntropy’ seems to be the most irrelevant to the ‘legitimate’ feature. Thus, Figure 3 is a Multivariate Analysis visualization of the eight most important features, as those were selected by the SelectKBest and the Extra Trees Classifier methods applied.

### 3.3. GANs Architecture

Nowadays, the situation of lacking enough data for training neural network models is often encountered. Ian Goodfellow et al. introduced, in 2014 [31], a deep learning architecture, namely Generative Adversarial Networks (GANs) destined to address this issue. The application of these advanced deep learning techniques is intended to generate multivariate types of synthetic datasets, including images, tabular data, texts, videos, and music composition, featuring a high similarity degree to the original ones.

Generative Adversarial Networks (GANs) modelling is defined as an architectural approach that provides and extends the ability of generating new, synthetic datasets, based on the specified input data as well as on random noise. This study focuses on such a GAN architecture, which consists of two deep neural network components that behave in an adversarial manner to each other, namely the generator and the discriminator. The generator is a deep neural network that receives as input a vector of random numbers, indicated as random noise, and its main task is the generation of high quality, realistic data, similar to the provided content. The generated data samples are fed as input to the discriminator, alongside with a random sample from the initial dataset. On the other hand, the discriminator is implemented as a sequential deep neural network composed of dense and dropout layers, that classifies the input samples of data, as real (original) or fake (generated). The effectiveness of the discriminator (to classify real from fake data samples) is finally used to update and optimize the overall performance of the GAN (in other words the performance of both the generator and the discriminator).

In the current study, the Zero -Day GAN (ZDGAN) model architecture, that was developed in order to generate 1D (one dimensional) synthetic data from the previously described dataset (Section 3.1 and Section 3.2), was implemented on Tensorflow 2.0 [32] and made use of the high-level Keras API [33]. Table 2 depicts the output of the generator model architecture:

The sequential API was used to create a sequence object in which the different layers of the proposed deep neural network were stacked. The generator part of the proposed ZDGAN architecture consists of (i) the input layer that accepts the random generated noise scaled to the desired size, (ii) nine hidden layers activated with the ‘ReLU’ function and (iii) the output layer that is activated by the ‘linear’ function and whose dimension is the same as the dimension of the preprocessed dataset, i.e., equal to nine feature columns, as described in Section 3.2.

Following the description of the generator model, Table 3 defines the output of the discriminator model. The discriminator is also a simple, straightforward sequential model including four dense layers. The first three layers are activated by the ‘ReLU’ function whilst the output layer is activated by the ‘sigmoid’ function since it distinguishes the input samples to real (True) or fake (False). Additionally, a dropout rate of 20% was applied to both the visible (or input) and the two (2) hidden layers of the discriminator model.

**Figure 4:**
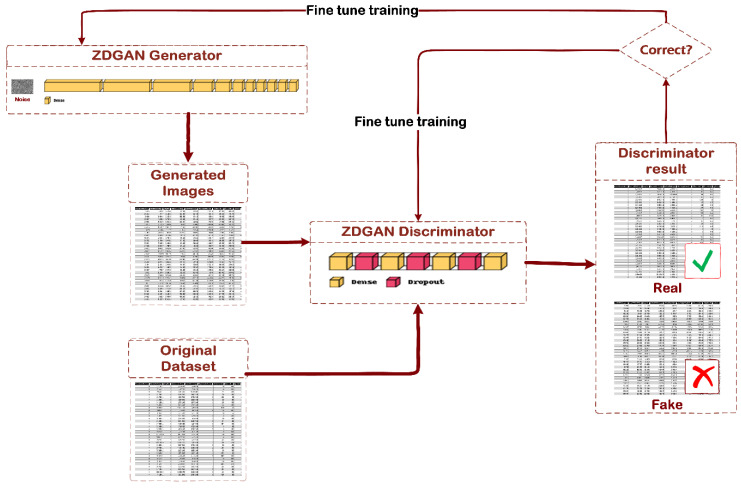
ZDGAN implementation.

After the description of the generator and the discriminator models, the proposed ZDGAN model is defined. This could be also characterized as a sequential model that combines the generator with the discriminator functionalities in an adversarial way. Figure 4 depicts the generation process of the preprocessed zero-day data, using the proposed ZDGAN for synthetic, tabular datasets.

### 3.4. GAN Implementation and Data Generation Results

In the previous paragraph, both the generator and the discriminator models were defined, so as to compose the overall proposed ZDGAN model. Afterwards, the training procedure was performed in order to proceed with the generation of datasets similar to the original ones. The training process was configured for 5000 epochs in total. For each epoch, a batch training of predefined size is performed on both the generator and the discriminator network. The discriminator accepts as input a predefined batch of data samples from the dataset as described in Section 3.2 along with the generated output data sample from the generator and computes the discriminator loss for both the real and the generated images. The loss values are, first, calculated separately and then are combined to form a min-max equation [31]. Equation (1) describes this min-max game where G is the Generator, D is the discriminator and $V\left(D,G\right)$ is the value function of the min-max game.

(1) $$min_{G}max_{D}V\left(D,G\right)=E_{x∼p_{g}\left(x\right)}\left[logD\left(x\right)\right]+E_{z∼p_{z}\left(z\right)}\left[log\left(1-D\left(G\left(z\right)\right)\right)\right]$$

Equation (1) can be applied to the ZDGAN of this study as it consists of straightforward multilayer perceptrons. As soon as the variables $p_{z}\left(z\right)$ for the input noise are “learned”, then the generator’s distribution $p_{g}\left(x\right)$ over data x can be learned too. Following this, the representation of the mapping to data space as $G\left(z\right)\;$ takes place, where G is a differentiable function represented by a multilayer perceptron with parameters *θ_g_*. Afterwards, a second multilayer perceptron $D\left(x\right)\;$) is defined which outputs a single scalar as described previously. $D\left(x\right)\;$ represents the probability that x originated from the data rather than the generator’s distribution *p_g_*. Finally, the discriminator is trained to maximize the probability of assigning the correct label to both the original and generated images and samples, whilst the generator is trained to minimize $log\left(1-D\left(G\left(z\right)\right)\right)$.

To this end, the discriminator losses improve the discriminator model’s exposed predictions and lead to the computation of the generator losses and the gradient, because of the employment of backpropagation processes. However, the generator continues to improve the synthetically (in tabular format) generated data samples, through the continuous updates of the generator weights using these gradients.

**Figure 5:**
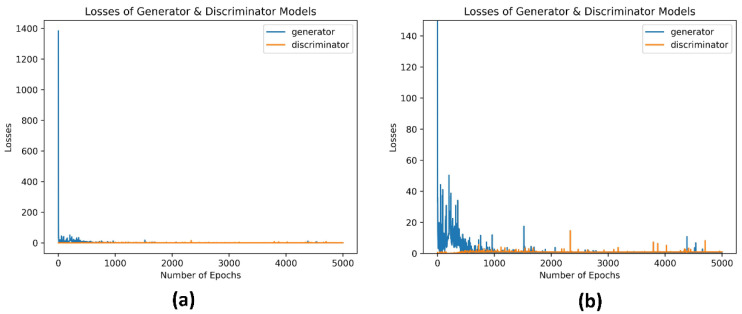
(**a**) Generator and discriminator losses full scale. (**b**) Generator and discriminator losses zoomed in scale 0–150.

As becomes apparent from Figure 5, the losses are saturating after a certain point of the executed iterations, during the generation process of zero-day (malware-based data in tabular format) samples, both for the generator and the discriminator. More specifically, the generator loss started quite high, close to 1400, whilst the discriminator loss was considerably lower, approximately 0.667. This was an expected result, as in the beginning of the training process, the generator could not produce synthetic tabular data similar to the real ones. Hence, the discriminator could, quite easily classify them correctly. As the training process progressed, the generator loss started to reduce, taking values from 665 to 6.5, spanning the iterations 2 to 10, and between 0.544 and 4.897 for the iterations from 11 to 590. At this point, it is worth mentioning that, even though the generator seems to be stabilized after the iteration 1500, nonetheless some (loss) spikes are observed until the end of the training process (e.g., at the 4385th iteration, where is equal to 10.761). This phenomenon could be characterized as an isolated failure of the model, during the training process, due to the random noise introduced to it, in order to generate random samples. During the same iteration intervals, the discriminator loss increased since it was no longer as easy to correctly classify the synthetic tabular datasets with high probability.

After the completion of the ZDGAN training process, the result of this adversarial procedure leads to the creation of a robust generator model, whose generated synthetic data samples in tabular format are quite difficult to be classified as original or generated by the discriminator model.

**Figure 6:**
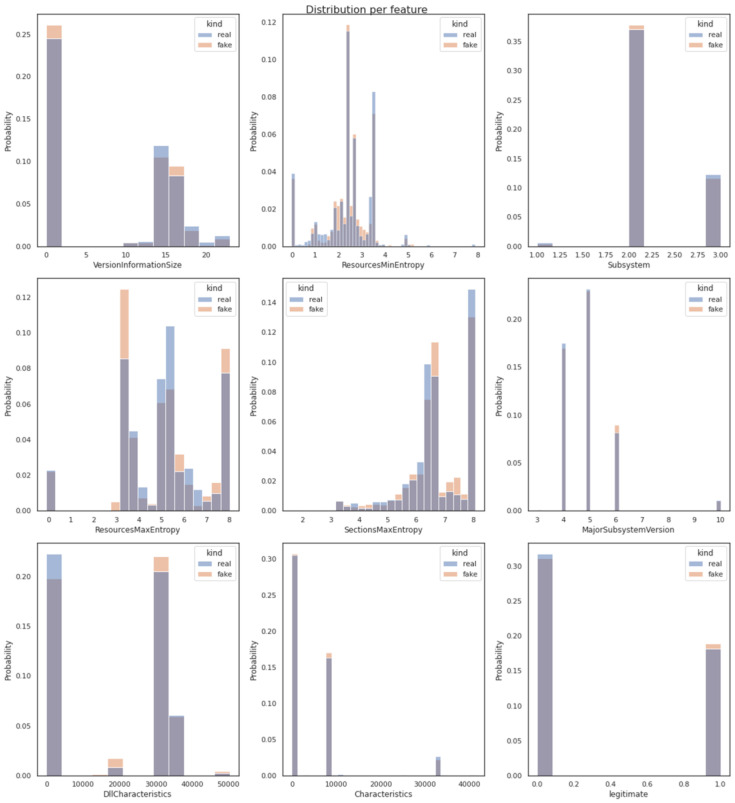
Similarity of real and fake data samples between pair variables using distribution metrics techniques.

Apart from developing powerful and similar-looking synthetic data using the previously described cutting-edge technique, one of the most important and challenging parts of this study was the choice of the similarity evaluation metrics, so as to evaluate whether the synthetic, generated data were structurally and statistically similar to the original data. Firstly, the similarity between these tabular data (namely real and fake data in our study) was measured for each pair-columns independently. Afterwards, the relationships across multiple variables (columns) in each dataset were measured. Figure 6 determines the similarity between pair variables using Distribution Metrics techniques. The statistical similarity between the pair variables of the real (original) and the fake (generated) dataset was calculated by evaluating their probability distributions. More specifically, following the extraction of nine histogram visualizations (one for each of the dataset’s features as described in Section 3.2 and one for the target data label-legitimate) of pair variables in the original and the generated dataset, it became clear that the depicted probability distributions were in the same range for both the synthetic data and the original dataset.

**Figure 7:**
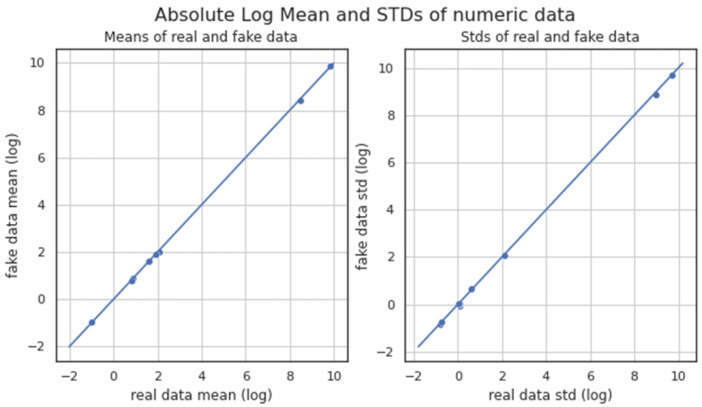
Absolute, log, mean and STD of original (real) and generated (fake) data.

Figure 7 depicts the absolute mean and the standard deviation (STD) of numerical data between the original and the generated dataset. Both the mean absolute deviation and the STD are a measure of variability, providing information on how spread out considered datasets are. Figure 7 indicates that the data values are very close to the mean and the STD, since the distance of each data point to these two statistical points seems quite small. Thus, the depicted mean absolute and STD distributions of the original and generated datasets are (almost) overlapping, therefore allowing us to deduce that the spread of each corresponding dataset was similar, while also being most likely that the two datasets are quite close to each other form a statistics viewpoint.

**Figure 8:**
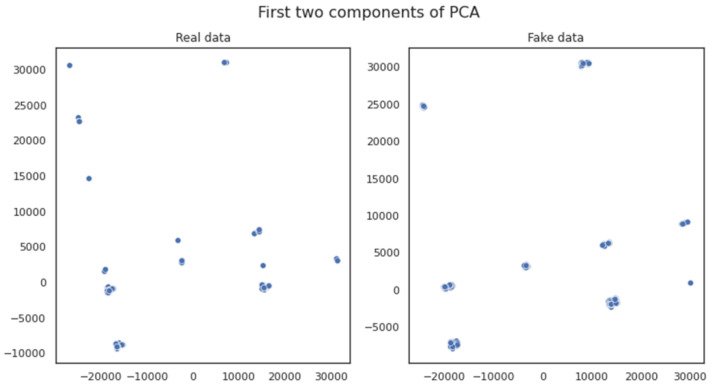
Visualization of the first two components of the PCA analysis.

Figure 8 visualizes the first two components of the Principal Component Analysis (PCA), a linear feature extraction technique for performing a linear mapping of the data to a lower-dimensional space in such a way that the variance of the data in the low-dimensional representation is maximized. The distributions featured in Figure 8 indicate that the observed correlation in the depicted PCA dimensions is quite high between the variables of the original and the generated dataset. Based on the fact that a small number of uncorrelated variables are observed, this could lead to the extraction of similar conclusions regarding the data properties (data features) of both the original and the generated datasets, which feature a high degree of similarity and (feature) correlation between each other.

**Figure 9:**
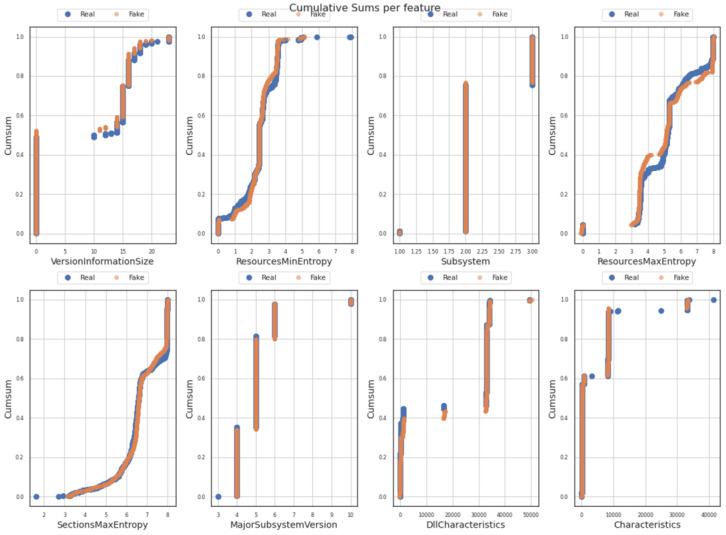
Visualization of cumulative sums for each of eight features of the original (real) and generated (fake) dataset.

Figure 9 is composed of eight sub-figures that depict the cumulative sums per feature (one for each of the eight independent/input data features mentioned in Section 3.2). A cumulative sum chart can be used to detect and visualize possible deviations between the original and the generated values as well as small shifts in their mean values (of the target/original values), collected at different time intervals. Following the results depicted in the (sub)figures below, no high or important deviation between the process mean (generated dataset) and the target mean (original dataset), for each of the eight (8) features was observed. The results provide valuable information on the degree of similarity (which is indicated as adequately high) for trends, patterns, and thresholds over time, both on the original and the generated data features.

**Figure 10:**
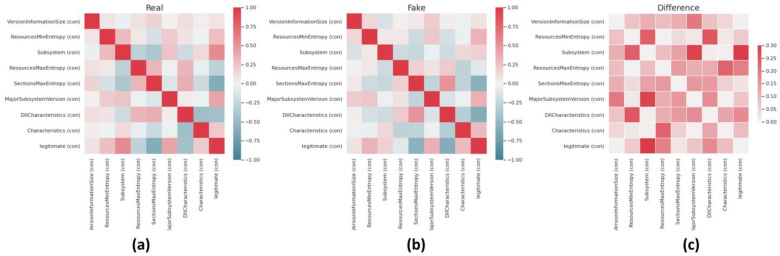
Correlation differences between features in original (**a**), generated (**b**) and between them (**c**).

Finally, Figure 10 visualizes the correlation differences between all the possible pairs of values in the original (left) and generated (middle) as well as the actual difference (right) between them, using correlation matrixes with heatmap(s). As can be suggested by the correlation matrix of Figure 10, correlation coefficients whose magnitude are between 0.2 and 1.3 indicate data variables which can be considered very highly correlated. However, correlation coefficients whose magnitude is between 0.1 and 0.2 indicate variables that can be considered highly correlated, whereas the ones whose magnitude is between 0.01 and 0.1 indicate variables that can be considered as moderately correlated. Following the analysis results depicted in the Correlation Matrices of Figure 10, the high similarity between all possible pairs of the compared dataset features is clear.

## 4. Zero-Day Attacks Classification Using Neural Networks

The effectiveness and the overall contribution of the generated samples produced, as described in Section 3.4, are further assessed by evaluating a Deep Learning classifier. For these evaluation purposes, the DL Neural Network classifier was trained firstly with the original dataset as presented in 3.2 and then with the mixed dataset which contains both the original and the generated samples. It is worth mentioning that the DL model designed and studied was not the optimal one (in terms of accuracy and loss), since the study focuses more on the effectiveness of the data generated via the GANs, than on the performance of the (presented) deep learning model. Table 4 summarizes the size of the training and evaluation samples for the three different datasets used.

Additionally, indicative metrics about the training process of the NN classifier are depicted in Table 5. More specifically, the precision, recall, the F1-score and the support metrics for each class (0 and 1) are presented. As it is obvious, the ‘Recall’ indicator, the proportion of actual positive data instances which were correctly predicted, is equal in both legitimate and non-legitimate label predictions for the two models. On the other hand, the results of the ‘Precision’ indicator, the proportion of positive cases of data instances that were successfully predicted, presented a variation. In both models, the computed precision values were slightly higher for the non-legitimate (zero values), than for the legitimate values (one’s values). This occurred due to the fact that it was much easier for the model to successfully predict the non-legitimate data instances. Finally, the ‘F1-score’ indicator, since it is computed as a combination result of the precision and recall metrics, revealed the same type of fluctuations, as expected.

Furthermore, Table 6 depicts False Positives (FP), False Negatives (FN) and Misclassification rates, metrics which were derived from the confusion matrix of each model respectively. The Confusion matrix is a special kind of contingency table suitable for visualizing the performance of the predicted model. Each entry in a confusion matrix denotes the number of predictions classified by the model correctly or incorrectly. False Positives (FP) refer to the number of predictions where the trained model incorrectly predicts the negative classes as positives, whereas False Negatives (FN) refer to the number of predictions, where the positive classes are predicted as negatives incorrectly. The Misclassification Rate is a performance metric used to evaluate the performance of the trained model. This metric, also known as Classification Error or Error Rate (metric), provides the fraction of predictions that are incorrectly classified by the model. It is also worth noting that the metrics depicted in Table 6 are only used to evaluate the performance of the model and are irrelevant to the loss function [34]. The last parameter (loss function) is used to optimize the performance of the model, after its continuous effort to minimize the model’s losses during the training loop procedure [35]. The loss function that was selected during the training procedures of our models was the ‘categorical_crossentropy’.

After the completion of both training procedures, the two models were evaluated by providing them the original dataset, the generated dataset and a combined dataset which contained both original and generated samples as input. The accuracy, validation loss results, as well as the False Positives (FP), False Negatives (FN), and Misclassification Metrics, of these six (three and three) evaluations phases are summarized in Table 6 and Table 7.

These results indicate the effectiveness and the contribution of the generated dataset and how this can lift data limitations in such sensitive applications such as zero-day attacks. As it is obvious, the model that was trained with solely the original dataset was efficient when it was evaluated only using data from the original dataset. However, its performance in the combined dataset was quite poor, especially, in terms of loss compared to every other scenario. On the contrary, the model trained with the combined dataset achieved remarkable results when evaluated with a combined dataset but the results for other evaluation datasets are, also, better in terms of the loss metric. Additionally, as far as the misclassification rate is concerned, the model that was trained with the combined dataset performed slightly better overall. Taking into account all of the results featured above, the significance of data generation using cutting edge technologies, like GANs, in order to create and provide larger and better datasets in an era where data are considered to be very valuable and the lack of them consists a major limitation of Artificial Intelligence applications, is documented. Such data inadequacy is the consequence of data privacy policies around the world which have hindered data from being shared and used efficiently for further analytics and innovation. The goal and the added value of this study is to solve this conflict between data privacy and data availability.

## 5. Conclusions

The present study aimed to describe a complete methodology for synthetic zero-day attacks data generation using GANs as well as to highlight the significance of such generation by comparing the same Neural Network classifier trained with and without generated samples. The data generation process engaged a Kaggle’s dataset which contains the raw data that had been obtained from the malware security partner of Meraz’18, the annual techno-cultural festival of IIT Bhiali. Based on these tabular format data, the ZDGAN architecture, that was presented in detail in Section 3, produced 200,000 new zero-day data samples which were almost identical to the original.

In order to evaluate the added value of the proposed data generation process using GANs, a Neural Network classifier was evaluated by using different training inputs. Specifically, the NN classifier, presented in Section 4, was trained using three different zero-day datasets: (i) the original dataset from Kaggle, (ii) the generated samples dataset and (iii) a combination of the two aforementioned datasets. The accuracy and loss results indicated that the performance of the NN classifier was better when the large dataset, which was produced from the combination of the original and the generated datasets, was used. This indicated that through the suggested data generation process using GANs, currently existing datasets can be expanded in order to improve the training process of DL algorithms. This is very important for cybersecurity experts as it is very difficult to have large, high-quality datasets especially related to zero-day attacks. Thus, the use of GANs for creating data samples can result to the production of new large datasets which can then be used to efficiently train DL classifiers to achieve better results in terms of zero-day detection and classification of eminent zero-day threats.

The future prospect of this study is to expand the categories and the generated data domains into other domains, different data formats as well as other cyberthreats. Moreover, the exploration of lifelong learning techniques both for the data generation as well as for zero-day detection and classification of zero-day attacks is a future step towards this direction. Additionally, by engaging lifelong learning techniques, newly discovered data from real-world zero-day attacks can be fed to the ZDGAN in order for the later to learn new patterns and adjust accordingly the generator results in an endless loop of training-generation-evaluation. This will lead to constantly updated datasets and trained DL algorithms which will not only be able to detect but also to predict possible zero-day vulnerabilities or attacks for newly launched software solutions.

## Figures and Tables

**Table 1:** Top 30 features based on their importance score.

Feature	Score
SectionsMaxEntropy	9.11329 × 10^13^
Subsystem	6.35191 × 10^14^
DllCharacteristics	4.89078 × 10^14^
MajorSubsystemVersion	4.449 × 10^14^
ResourcesMinEntropy	2.06557 × 10^14^
VersionInformationSize	1.80669 × 10^14^
ResourcesMaxEntropy	1.63419 × 10^14^
Characteristics	1.18211 × 10^14^
CheckSum	6.87253 × 10^13^
SectionsNb	5.43979 × 10^14^
MinorLinkerVersion	3.33103 × 10^14^
md5	2.65074 × 10^14^
ExportNb	2.10984 × 10^14^
SectionsMeanEntropy	1.95062 × 10^14^
SectionsMinEntropy	1.92938 × 10^14^
ImportsNbOrdinal	1.688 × 10^14^
ResourcesMeanEntropy	1.55226 × 10^14^
ResourcesNb	1.05029 × 10^14^
MajorImageVersion	9.87539 × 10^14^
MinorImageVersion	8.74982 × 10^14^
SectionsMinRawsize	6.96936 × 10^13^
ImportsNb	6.2723 × 10^14^
SectionsMinVirtualsize	6.13542 × 10^14^
SizeOfImage	8.43647 × 10^14^
FileAlignment	6.20204 × 10^14^
LoadConfigurationSize	5.33371 × 10^14^
SizeOfHeaders	4.91498 × 10^14^
SizeOfUninitializedData	3.80247 × 10^14^
MajorLinkerVersion	1.85671 × 10^14^
SectionMaxVirtualsize	1.68149 × 10^14^

**Table 2:** Generator model output.

Generator		
Layer (Type)	Output Shape	Number of Parameters
dense (Dense)	(None, 1536)	13,824
dense_1 (Dense)	(None, 1278)	1,964,286
dense_2 (Dense)	(None, 1024)	1,309,696
dense_3 (Dense)	(None, 512)	524,800
dense_4 (Dense)	(None, 384)	196,992
dense_5 (Dense)	(None, 256)	98,560
dense_6 (Dense)	(None, 128)	32,896
dense_7 (Dense)	(None, 64)	8256
dense_8 (Dense)	(None, 32)	2080
dense_9 (Dense)	(None, 16)	528
dense_10 (Dense)	(None, 9)	153
Total parameters	4,152,071	
Trainable parameters	4,152,071	
Non-trainable parameters	0	

**Table 3:** Discriminator model output.

Discriminator		
Layer (Type)	Output Shape	Number of Parameters
dense_11 (Dense)	(None, 128)	1280
dropout (Dropout)	(None, 128)	0
dense_12 (Dense)	(None, 64)	8256
dropout_1 (Dropout)	(None, 64)	0
dense_13 (Dense)	(None, 32)	2080
dropout_2 (Dropout)	(None, 32)	0
dense_14 (Dense)	(None, 1)	33
Total parameters	11,649	
Trainable parameters	11,649	
Non-trainable parameters	0	

**Table 4:** Training, evaluation, and total samples of original, generated and combined dataset.

Training/ Evaluation Dataset	Original	Generated	Combined
Training samples	173,080	160,000	333,080
Evaluation samples	43,271	40,000	83,271
Total samples	216,351	200,000	416,351

**Table 5:** Training metrics for both training processes of the NN classifier.

Model (Training Dataset)	Label	Precision	Recall	F1-Score	Support
Original	0	0.98	0.96	0.97	28,170
	1	0.93	0.96	0.94	15,101
Combined	0	0.97	0.96	0.97	49,855
	1	0.96	0.96	0.96	33,416

**Table 6:** False Positives (FP), False Negatives (FN) and Misclassification Rates for the original and combined datasets.

Training/ Evaluation Dataset	Original		Generated		Combined	
	FP Rate	FN Rate	FP Rate	FN Rate	FP Rate	FN Rate
Original	1047	677	1858	2173	24,990	5376
Combined	3573	2767	7501	3724	1503	1507
Misclassification Rate	Original	Combined	Original	Combined	Original	Combined
	0.0398	0.1456	0.1008	0.2806	0.3647	0.036

**Table 7:** Accuracy and loss results for different trained model of the NN classifier and three different evaluation datasets.

Training/ Evaluation Dataset	Original		Generated		Combined	
	Accuracy	Loss	Accuracy	Loss	Accuracy	Loss
Original	0.9602	0.1266	0.7642	0.2935	0.6334	0.6646
Combined	0.8535	0.1184	0.7194	0.1689	0.9642	0.1184

## Data Availability

The data presented in this study are available on request from the corresponding author.

## Appendix A

**Table A1:** Initial dataset features and their data types.

**Feature**	**Data Type**
ID	int64
md5	object
Machine	object
SizeOfOptionalHeader	int64
Characteristics	int64
MajorLinkerVersion	float64
MinorLinkerVersion	int64
SizeOfCode	int64
SizeOfInitializedData	int64
SizeOfUninitializedData	int64
AddressOfEntryPoint	int64
BaseOfCode	int64
BaseOfData	int64
ImageBase	float64
SectionAlignment	int64
FileAlignment	int64
MajorOperatingSystemVersion	int64
MinorOperatingSystemVersion	int64
MajorImageVersion	int64
MinorImageVersion	int64
MajorSubsystemVersion	int64
MinorSubsystemVersion	int64
SizeOfImage	int64
SizeOfHeaders	int64
CheckSum	int64
Subsystem	int64
DllCharacteristics	int64
SizeOfStackReserve	int64
SizeOfStackCommit	int64
SizeOfHeapReserve	int64
